# Chemokine-Like Factor-Like MARVEL Transmembrane Domain-Containing Family in Hepatocellular Carcinoma: Latest Advances

**DOI:** 10.3389/fonc.2020.595973

**Published:** 2020-11-13

**Authors:** Mengxia Li, Fangzhou Luo, Xinyao Tian, Shengyong Yin, Lin Zhou, Shusen Zheng

**Affiliations:** ^1^Division of Hepatobiliary and Pancreatic Surgery, Department of Surgery, The First Affiliated Hospital, Zhejiang University School of Medicine, Hangzhou, China; ^2^School of Medicine, Zhejiang University, Hangzhou, China; ^3^NHC Key Laboratory of Combined Multi-organ Transplantation, Hangzhou, China; ^4^Key Laboratory of the Diagnosis and Treatment of Organ Transplantation, Research Unit of Collaborative Diagnosis and Treatment For Hepatobiliary and Pancreatic Cancer, Chinese Academy of Medical Sciences (2019RU019), Hangzhou, China; ^5^Key Laboratory of Organ Transplantation, Research Center for Diagnosis and Treatment of Hepatobiliary Diseases, Hangzhou, China

**Keywords:** chemokine-like factor–like MARVEL transmembrane domain-containing family, chemokine-like factor, hepatocellular carcinoma, MARVEL, tumor suppressor gene

## Abstract

Chemokine-like factor (CKLF)–like MARVEL transmembrane domain-containing family (CMTMs) is a new gene family, consisting of CKLF and CMTM1 to CMTM8, which plays an important role in hematopoiesis system, autoimmune diseases, male reproduction etc. Abnormal expression of CMTMs is also associated with tumor genesis, development and metastasis. In this review, we briefly describe the characteristics of CMTM family, outline its functions in multiple kinds of carcinomas, and summarize the latest research on their roles in hepatocellular carcinoma which are mainly related to the expression, prognostic effect, potential functions, and mechanism of action. The CMTM family is expected to provide new ideas and targets for HCC diagnosis and treatment.

## Introduction

Chemokine-like factor (CKLF)–like MARVEL transmembrane domain-containing family (CMTMs) as a new gene family, consists of nine genes totally, which includes CKLF and CMTM1 until CMTM8 genes. CKLF1 and its three variants were first discovered and reported by Han et al. from Peking University Human Disease Gene Research Center in 2001 (1). They also identified CMTM1 to CMTM8 genes by reverse transcription polymerase chain reaction (PCR) techniques in the subsequent studies (1, 2). The genes of CMTM families are located on different chromosomes. CKLF and CMTM1–4 form a gene cluster on chromosome 16q, CMTM5 is located on chromosome 14q11.2, and CMTM6–8 form another gene cluster on chromosome 3p22.3 (2). They have different alternative RNA splicing forms respectively. Their coding products are mostly distributed both in the cytomembrane and cytoplasm. The functional characteristics of the gene products lie between classical chemokines and members of the transmembrane 4 super family (TM4SF). These characteristics partly owe to the special molecular structure of CMTMs protein which contains the MARVEL domain with four transmembrane-helix architecture and is closely linked with vesicle transport and membrane binding related events (3–5).

The members of CMTM family are widely expressed in human tissues and involve multiple biological systems, such as immune (5–9), male reproductive (10–12), hematopoiesis (13, 14), circulatory (15, 16), and muscular systems (1, 17). The abnormal expressions of CMTMs are associated with various diseases. CKLF1 mediates the immune inflammatory reaction process in rheumatoid arthritis and atopic dermatitis (18, 19). CKLF1 helps to promote the migration and proliferation of vascular smooth muscle and skeletal muscle cells (1, 17). Additionally, CKLF1 is also a potential target for the treatment of focal cerebral ischemia and cardiopulmonary complications (16, 20). CMTM1, CMTM2, CMTM3, and CMTM4 play a crucial role in the spermatogenesis process or testicular development, and these CMTMs could be used as potential molecular markers for diagnosis or treatment of male infertility (10–12, 21). CMTM2 exhibits a negative regulatory effect on human immunodeficiency virus type-1 transcription by inhibiting the AP-1 and CREB pathways (22). The promoter hypermethylation of CMTM2 could distinguish Sézary syndrome from erythrodermic inflammatory dermatosis (7). Both CMTM3 and CMTM4 are associated with angiogenesis by regulating cell surface availability of VE-cadherin (13, 14). Methylation levels in CMTM4 and CMTM5 are significantly different in the case of systemic lupus erythematosus and rheumatoid arthritis (8). CMTM7 contributes to B-1a cell development where the regulation of B-cell antigen receptors expression takes place (23). The polymorphisms of CMTM7 gene are associated with heart failure mortality among the European patients and increase the risk of obesity in Han Chinese male children (15, 24). CMTM8 promotes bone marrow-derived mesenchymal stem/stromal cells proliferation and migration *via* the epidermal growth factor receptor (EGFR) signaling, which provides a new research direction in bone regeneration and tissue engineering (25).

With the in-depth study, researchers found that the CMTMs do not only play important biological roles in these diseases as mentioned above, but also have been implicated in various cancers, involving tumorigenesis, development and metastasis (26). Different CMTMs have different effects on tumors, some of them have become potential therapeutic targets or prognostic indicators of tumors. Hepatocellular Carcinoma (HCC) is one of the most common malignancies worldwide with high recurrence rate and poor prognosis. Alpha-fetoprotein is the main serum biomarkers to diagnosis of HCC over past years, but the specificity and sensitivity is not satisfactory. There is a pressing need to explore novel biomarkers to detect early onset HCC and identify prognostic biomarkers for HCC to improve clinical outcomes. In addition, effective molecular therapeutic target is lacking which limit the roles of molecular targeted therapy and immunotherapy in HCC. Recent researches have revealed that CMTMs might also be closely associated with HCC. CMTM members expected to represent promising targets for HCC diagnosis and treatment. Herein, we briefly describe the characteristics of the CMTM family, outline the functions of CMTM family in diverse tumors, and focus on summarizing the latest studies on the detailed functions and underlying molecular mechanisms of CMTMs in HCC (**Table 1**).

**Table 1:** CMTMs functions in Hepatocellular Carcinoma.

CMTMs	Protein type	Pro-/anti-tumor	Mechanism	Effects on HCC cells *in vitro*/xenograft tumor model	Clinical significance	Ref.
CKLF1	Secreted/Transmembrane protein	pro-tumor	Activates the IL-6/JAK/STAT3 signaling pathway	Promotes cells proliferation, migration and invasion, prevent Doxorubicin-induced apoptosis/Promote carcinogenesis and progression in in Balb/c nude mice.	Highly expressed in HCC clinical samples and was an independent risk factor for poor survival.	Liu et al. (27)
CMTM1	Secreted/Transmembrane protein	NA	NA	NA	the expression level of CMTM1-v17 mRNA was high in liver cancer.	Wang et al. (28)
CMTM2	Secreted/Transmembrane protein	anti-tumor	Inhibits EMT process, positive correlation with E-cadherin	Inhibits cells proliferation, invasion and migration.	Down-regulated in HCC tissues and was an independent protective factor for the prognosis of HCC patients.	Guo et al. (29); Zhang et al. (30)
CMTM3	Secreted/Transmembrane protein	anti-tumor	suppresses the JAK2/STAT3 signaling pathway	Inhibits the proliferation, invasion, and EMT process in HepG2 cells/Attenuates the tumor growth in Balb/c nude mice.	Act as a putative tumor suppressor in the development and progression of HCC	Li et al. (31)
CMTM4	Transmembrane protein	anti-tumor	NA	NA	Down-regulated in HCC tissues and the negative expression was a risk factor for poor prognosis of HCC.	Bei et al. (32)
		pro-tumor	NA	NA	Increased CMTM4 mRNA expression predicted an unfavorable prognosis of HCC.	Zhou et al. (33)
CMTM5	Secreted/Transmembrane protein	anti-tumor	Suppresses PI3K/AKT signalling pathway	Inhibits cell growth, promote cell apoptosis, and reduce cell metastatic and invasion/Suppresses xenograft tumor growth *in vivo*.	Down-regulated in HCC tissues and was associated with poor prognosis.	Xu et al. (34)
		pro-tumor	NA	NA	CMTM5 SNP rs3811178 might contribute to the genetic susceptibility of HCC.	Bei et al. (35)
CMTM6	Transmembrane protein	anti-tumor	NA	NA	Down-regulated in HCC tissues, and correlated with HCC metastasis and survival of HCC patients.	Zhu et al. (36)
		pro-tumor	Suppresses T-cell by stabilizing PD-L1 in the membrane	Up-regulates PD-L1 on the hepatocyte surface.	CMTM6 SNP rs164207 might contribute to the genetic susceptibility of HCC. CMTM6/PD-L1 coexpression was associated with poorer survival rate in HCC patients.	Yamamoto et al. (37); Bei et al. (35); Liu et al. (38)
CMTM7	Transmembrane protein	anti-tumor	Suppresses PI3K/AKT signaling and induce cell cycle arrest at the G0/G1 phase	Inhibits the cell growth and migration.	Down-regulated in HCC tissues and negatively correlated with TNM staging and tumor metastasis.	Huang et al. (39)
CMTM8	Transmembrane protein	anti-tumor	Inhibits c-MET/ERK signaling pathway	Inhibits hepatocyte growth factor-induced EMT-like morphological changes and cell motility.	NA	Zhang et al. (40)

CMTM, Chemokine-like factor (CKLF)–like MARVEL transmembrane domain-containing; EMT, epithelial-mesenchymal transition; SNP, single-nucleotide polymorphisms; NA, not available.

## CMTM Family Members and HCC

### CKLF

**Figure 1:**
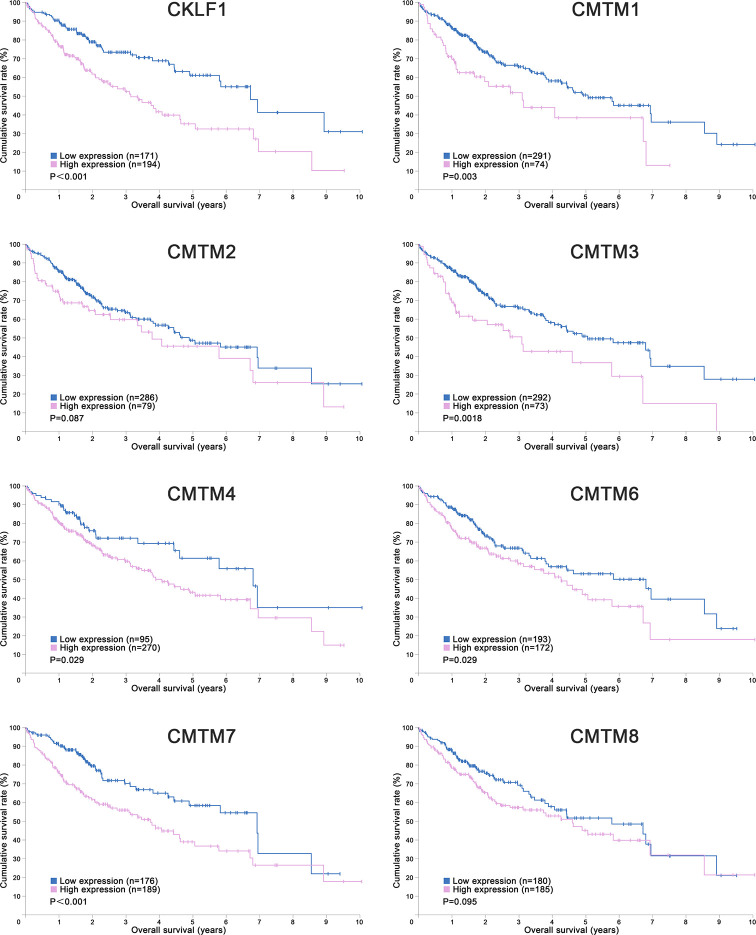
Kaplan-Meier Curves of CMTMs in Hepatocellular Carcinoma. Image credit: the Human Protein Atlas database. https://www.proteinatlas.org/.

CKLF is the first identified member of CMTM family which located at chromosome 16q21. It has at least four alternative RNA splicing forms: CKLF1, CKLF2, CKLF3, CKLF4, in which CKLF2 is a full length cDNA product (1). CKLF1 and CKLF3 are secreted isoform, whereas CKLF2 and CKLF4 are transmembrane isoform. CKLFs show the most similarity to chemokines in CMTM family. These proteins have broad-spectrum chemotactic activity by interaction with human CC chemokine receptor 4 (CCR4) through two C-terminal peptides, C19 and C27 (41). Currently, a large number of studies have already revealed that CKLFs play an important role in inflammatory and autoimmune diseases. CKLFs may also have effects on both malignant and benign tumors (42). CKLF1 was upregulated in ovarian cancer tissues but downregulated in lung cancer tissues (42). Overexpressed CKLF1 was related to the formation of keloid and abdominal aortic aneurysms (43, 44). Recently, Liu et al. analyzed the expression level, prognostic value and potential function of CKLF1 in HCC (27). They found that CKLF1 was highly expressed in HCC tissues and related to the vascular invasion and tumor size. CKLF1 could activate the IL-6/STAT3 signaling pathway and up-regulation of Bcl-xl, MYC, and cyclins D1 to enhance HCC development and metastasis, and resist the apoptosis induced by Doxorubicin. Their findings were consistent with the results of the Human Protein Atlas which showed that the higher CMTM1 expression was, the poorer prognosis of HCC would be (**Figure 1**). Therefore, CKLF1 may be a pivotal modulator in the occurrence and development of HCC. CKLF1 could affect the biological behavior and prognosis of HCC, and is expected to become a potential target for the diagnosis and treatment of HCC.

### CMTM1

**Figure 2:**
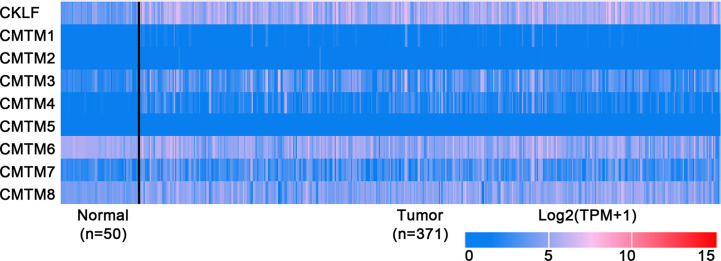
Expression profiles of CMTMs in Hepatocellular Carcinoma and the corresponding normal tissues. Image credit: UALCAN database. http://ualcan.path.uab.edu/.

CMTM1 is located at chromosome 16q21 and consists of 23 isoforms (CMTM1 v1-v23). The proteins of CMTM1 v1-16 and CMTM1 v17-23 are encoded by open reading frame 1 and open reading frame 2 respectively. The expression of CMTM1 is tissue specific and is predominantly expressed in testis tissue. Via PCR techniques, Wang et al. found that CMTM1-v17 was also highly expressed in many kinds of tumors, such as breast, kidney, lung, ovarian and liver cancers (28). The study demonstrated that CMTM1-v17 enhanced the cellular proliferation of breast cancer and prevented TNF-α-induced apoptosis by activating the NF-κB pathway. Si et al. discovered that CMTM1-v17 levels in non-small cell lung carcinoma groups was much higher than in parocarcinoma tissue, which might promote the chemoresistance and lead to poor prognosis (45). Additionally, CMTM1 promoted glioblastoma cells (A172 and U251MG) proliferation and invasion which have already been verified through *in vitro* experiments (46). Mays et al. found that CMTM1 was highly expressed in salivary adenoid cystic carcinoma (SACC) cells (SACC-83) (47). When the tumor tissues without recurrence/metastasis was compared with SACC-LM and tumor tissues with recurrence/metastasis, the result showed that CMTM1 might have enhanced the effectively of anti-tumor metastasis in SACC. A new study found that CMTM1-v5 specifically induced the human lymphoma cells apoptosis and may be a novel therapeutic for lymphoma treatment (48). Except for Wang et al. reported that CMTM1-v17 mRNA level was higher in liver tumor tissues than normal tissues which was consistent with the data from UALCAN (**Figure 2**), there was no other study has been taken up to investigate the relationship between CMTM1 and HCC so far. The Human Protein Atlas showed that the higher level of CMTM1 expression was associated with a lower survival probability in HCC (**Figure 1**). Combined with the results of PCR and bioinformatics analysis, we speculate that CMTM1 may serve as a potential tumor promoting role in HCC progression. The function and mechanism of CMTM1 in HCC should be elaborated *in vitro* and *in vivo* experiments in further study.

### CMTM2

CMTM2 gene is closely linked with and CMTM1 on chromosome 16q21, the distance between CMTM1 and CMTM2 is only 311 bp. They share a high level of amino acid sequence identity. Similar with CMTM1, the expression of CMTM2 is also high in the human testicular tissues, which can be secreted further into the seminiferous tubules (49). Moreover, the expression level of CMTM2 in SACC patients with tumor recurrence and perineural invasion is low too (47). Choi et al. evaluated 240 patients with diffuse-type gastric cancer. The study revealed that the patients with higher CMTM2 expression had a better overall survival (50). The Human Protein Atlas showed that CMTM2 is not a prognostic index in HCC (**Figure 1**), but several studies have shown CMTM2 had clinical significance in HCC. By using bioinformatics and immunohistochemistry (IHC) methods, Guo et al. found that the CMTM2 expression in HCC tissues is lower than the expression level in paired parocancerous tissues (29). In addition, its expression correlates with HCC pathological grades as well. They further confirmed that knockdown of CMTM2 *in vitro* significantly decreased the expression of E-cadherin and β-catenin and increased the expression of N-cadherin, Vimentin, ZEB1 and ZEB2. CMTM2 inhibited invasion and migration in HCC cells (Huh-7 and SMMC7721) by suppressing the epithelial-mesenchymal transition (EMT) process (30). In summary, CMTM2 plays tumor suppressive role and the expression level in tumor tissues could be a predictor of HCC, which could further guide postoperative treatment. Furthermore, the regulatory mechanisms of CMTM2 in HCC require further study which could provide a new direction for HCC treatment.

### CMTM3

CMTM3 and CMTM4 genes are closely located at the tumor suppressor locus 16q22.1. CMTM3 promoter contains a typical CpG island. Different from other members in the CMTM family, alternatively spliced transcript variants of CMTM3 contain different 5′ UTRs, but encode the same protein. The protein only owns three transmembrane regions, in which one form is a secretory protein released *via* exosomes (2, 51). CMTM3 showed tumor-suppressive function while the DNA methylation of CMTM3 promoter inhibited CMTM3 expression in multiple tumors, such as gastric, breast, nasopharyngeal (52), male laryngeal (53), esophageal (54), and colon carcinomas (55). Li et al. revealed that CMTM3 could potentially induce a G2 cell cycle arrest and apoptosis in a p53-independent manner, thereby suppressing the proliferation and migration capabilities of testicular cancer cell line (NCCIT) (56). High expression of CMTM3 might correlate with favorable prognosis in gastric cancer, which not only inhibits the EGF-mediated tumorigenicity by promoting Rab5 activity, but also suppresses metastasis of gastric cancer *via* the STAT3/Twist1/EMT pathway (57–59). High expression of CMTM3 in prostate cancer cell also showed anti-tumor effects but the specific molecular mechanism remains unclear (60). In contrast with the anti-tumor effect, CMTM3 showed cancer-promoting effects in glioblastomas. Delic et al. found that higher CMTM3 expression was significantly correlated with shorter overall survival in glioblastomas by analyzing a large clinically annotated dataset. Knockdown of CMTM3 significantly reduced proliferation and invasiveness in U251MG cells (46). In HCC research, Li et al. confirmed that the expression of CMTM3 in HCC cell lines (HepG2, 97H, Hep3B, and HCCLM3) was low, and CMTM3 inhibits the proliferation and metastasis of HCC cells partially *via* suppressing the JAK2/STAT3 signaling pathway (31). Moreover, Zhao et al. identified the different expression of chemokines and chemokine-related genes between subsets of myeloid-derived suppressor, and put forward that CMTM3 may participate in regulating the tumor microenvironment of HCC (61). However, the results of the UALCAN and the Human Protein Atlas showed that higher CMTM3 expression was detected in liver tumor tissues and associated with poor prognosis, which is not consistent with the current researches (**Figures 1** and **2**). The relationship between CMTM3 expression and clinicopathological features of HCC patients is not clear yet. Therefore, more studies are needed to elucidate the clinical significance of CMTM3 in HCC diagnosis and prognosis from clinical samples.

### CMTM4

CMTM4 is the most conserved member in CMTM family, and consists of three transcript variants including CMTM4-v1, CMTM4-v2, and CMTM4-v3. The CMTM4-v1 and -v2 are the main forms and widely expressed in multiple human tissue while on the other hand, the CMTM4-v3 only exists in certain renal and placental tissues (2). Kittler et al. successfully identified that CMTM4 was one of the 37 genes required for cell division in HeLa cells by using an endoribonuclease-prepared short interfering RNAs technique (62). The knockdown of CMTM4 caused the defect on cellular cleavage and resulted in bi-nucleated cells after mitosis. Plate et al. further studied the characteristics and functions of CMTM4 in HeLa cells. To the contrary, they found that CMTM4-v1 and -v2 inhibited HeLa cell growth by inducing G2/M phase accumulation without apoptosis (63). After that, many studies explored the expression and function of CMTM4 in a plenty of tumors, such as clear cell renal cell carcinoma (64), colorectal cancer (65) and pancreatic cancer (66). CMTM4 is frequently downregulated and act as a tumor-suppressor. The potential pathway mainly involved PI3K/AKT, ERK1/2, and STAT3 signaling pathways. Interestingly, Mezzadra et al. demonstrated that CMTM4 was a back-up regulator of programmed cell death ligand 1 (PD-L1) expression, which had the ability to protect PD-L1 protein from ubiquitination (67). Another research analyzed genes consistently overexpressed in 23 solid tumor types, and CMTM4 was identified as one of CD8^+^ T cell low tumor associated genes (68).

CMTM4 also showed clinical importance in liver cancer. Rong et al. found that CMTM4 was an oncogene in intrahepatic cholangiocarcinoma, and could be upregulated after knocking-down of FTO (69). In HCC, Bei et al. compared the expression of CMTM4 in cancer and parocancerous tissues using IHC (32). They concluded that there was a lower CMTM4 protein expression level in HCC tissues and further suggested that it might be a risk factor for poor prognosis in HCC patients. However, this result wasn’t consistent with the data obtained from the Human Protein Atlas (**Figure 1**) and UALCAN (**Figure 2**). Recently, by analyzing the Cancer Genome Atlas (TCGA) and Gene Expression Omnibus (GEO) database, Zhou et al. found the expression of CMTM4 mRNA copies were significantly upregulated in HCC tissues and correlated with poor prognosis (33). Furthermore, they discovered CMTM4 had negative correlations with immune cells in HCC, and proposed CMTM4 might play an important role in HCC immune microenvironment. To date, the difference of CMTM4 in protein and mRNA expression levels has not been well explored. Whether there is mutation, promoter methylation, posttranscriptional regulation, or microRNAs regulation of CMTM4 expression in HCC is unknown. In addition, the expression of CMTM4 in HCC cell lines has not been well studied, whether existing of difference between mRNA and protein expression is unclear. Considering that CMTM4 may be involved in cell cycle regulation and related to PD-L1 protein re-expression, the role of CMTM4 in immunotherapy for HCC worthy further investigation.

### CMTM5

CMTM5 is independently located at chromosome 14q11.2, and contains at least 6 alternatively spliced isoforms, ranging from CMTM5-v1 to -v6 (2). CMTM5-v1, as an evolutionarily conserved protein with 42% homology to CMTM3, is the major form in human CMTM5 and could be secreted *via* a vesicle mediated secretory pathway (51, 70). CMTM5 is highly expressed in human brain and blood (basophil, neutrophil), but frequently downregulated or silenced in pancreatic (71), cervical (72), oral (73), ovarian (74), prostate (75), renal (76), liver (34), and breast cancers (77). Similarity to the CMTM3 gene, the promoter methylation of CMTM5 gene is the main mechanism of tumor evading anticancer effect (78). By far, CMTM5 exhibits tumor suppressor activity in different types of cancer. For example, CMTM5-v1 induces pancreatic cancer cell lines (AsPC-1, BxPC-3, PANC-1, and MIA PaCa-2) apoptosis with activation of caspase 3, 8, and 9, and has synergistic effects with TNF-α (71). In renal cancer, restoration of CMTM5 could significantly inhibit ACHN cells growth by inducing cell cycle arrest in G0/G1 phase and apoptosis, and obviously suppress the cells migration and invasion (76). Another team confirmed that CMTM5-v1 is significantly downregulated in all the prostate cancer cell lines (PC3, DU 145, 22Rv1, and LNCaP). Ectopic expression of CMTM5-v1 could inhibit prostate cancer cell proliferation and migration by downregulating oncogenic EGFR signaling. Furthermore, CMTM5-v1 can improve the sensitivity of PC3 cells to Gefetinib, an EGFR tyrosine kinase inhibitor (79).

A case-control study indicated a strong correlation between rs3811178 in CMTM5 and risk of HCC in the southern Chinese population (35). Xu and Dang found CMTM5 was significantly reduced in 77.6% (59/76) of HCC tissues compared with the paired adjacent nontumor tissues, as well as in Huh7, Hep3B, HepG2, and SMMC-7721 cell lines. Overexpression of CMTM5 significantly inhibited Huh7 cell growth and metastasis *in vitro* and inhibited xenograft tumor growth *in vivo*. They further observed that CMTM5 is negatively correlate with the expression of PI3K, pAKT, Bcl2, cyclinD1, cyclinE, MMP2 and MMP9, and is positive correlation with the expression of p21, Bax, Bad, and cleaved caspase3 in HCC. The results suggested that CMTM5 may suppress HCC growth and metastasis through inhibiting PI3K-AKT signaling (34). In another study, Guan et al. proved that miR-10b-3p, acting as an oncogenic role, was dramatically up-regulation in HCC cell lines (HepG2) and the expression of CMTM5 was significantly suppressed (80). In addition, previous study indicated that the expression of CMTM5 in HCC could be restored by the treatment with PXD101, a histone deacetylase inhibitor (81). Taken together, CMTM5 may be expected to become a prognostic biomarker and valuable therapeutic target for HCC.

### CMTM6

CMTM6 is another member of CMTM family located at chromosome 3p22.3 where it shares 55% amino acid identity with CMTM4. The protein is broadly expressed in many normal tissues, mainly in the plasma membrane, but the exact function is still unknown. Recent studies identified that CMTM6 was co-localization with PD-L1 and acted as a critical regulator for the maintenance of PD-L1 expression in various cancer types (67, 82). By using a CRISPR–Cas9 deletion library screen, Burr et al. discovered that CMTM6 could protect internalized PD-L1 from lysosomal degradation and effectively recycled PD-L1 back to the cell surface (82). Through a haploid genetic screen, Mezzadra et al. demonstrated that CMTM6 could stabilize PD-L1 in the membrane by preventing ubiquitination, which then induced the T-cell suppression (67). Blocking the interaction of the two proteins by H1A, a PD-L1 antibody, results PD-L1 degradation (83). Both high expression of CMTM6 and PD-L1 were associated with better survival rate in breast (84) and lung cancer (85). CMTM6 overexpression could enhance the therapeutic effect of immune checkpoint inhibitors in advanced-stage non-small-cell lung cancer (86). In contrast, high CMTM6 expression was associated with poor prognosis for gliomas (87), pancreatic adenocarcinomas (84) and squamous cell carcinoma (88, 89). Interestingly, Zhao et al. found the effect of PD-L1 in renal cancer was bidirectional regulation by the CMTM6 level (90). High expression of PD-L1 will promote cancer progression when CMTM6 was overexpressed, but the effect will be reversed when CMTM6 was down-regulated.

In HCC, CMTM6 expression of mRNA in tumor samples was significantly lower than in normal samples (**Figure 2**). Zhu et al. compared the expression of CMTM6 in 75 paired HCC and adjacent nontumor tissues through IHC (36). They found that CMTM6 was in a lower expression level in HCC, and which correlated with tumor metastasis and low survival of patients. The confusing part is that the results of other studies are the opposite. Yafune et al. found that the protein level of CMTM6 in HCC was higher than that in non-tumor tissues (91). CMTM6 was co-expressed with CK8/18+, and may become a detection marker of hepatocyte proliferative lesions. Liu et al. demonstrated that CMTM6/PD-L1 coexpression was associated with poorer survival rate in HCC patients, especially macrotrabecular−massive HCC patients (38). The results of the Human Protein Atlas also showed that higher CMTM6 expression was associated with a poorer survival probability (**Figure 1**). Additionally, Bei et al. evaluated the genetic variant in CMTM6 with HCC risks, and found the individuals with rs164207 AA genotype have a higher risk of HCC than with CC genotype (35). Yamamoto et al. found that CMTM6 could be upregulated by anti-HBV drug Entecavir, then induced PD-L1 on the hepatocyte surface (37). The reasons for the discrepancy in the CMTM6 mRNA level and results of IHC between these studies are still not clear. Post-translation and posttranscriptional regulations are the possible factors. Small scale populations and observational bias may also influence the results. Given the participation of CMTM6 in the carcinogenesis of HCC and biological process of PD-L1 stabilization, combined treatment of anti-CMTM6 and anti-PD-L1 may be a new method to enhance the therapeutic benefits of immune checkpoint inhibitors in HCC.

### CMTM7

CMTM7 is located on the chromosome 3p22 which is rich in tumor-suppressor genes and the promoter contains a typical CpG island. The gene is evolutionarily conserved and encodes two isoforms, CMTM7-v1 and CMTM7-v2. CMTM7-v1 is the main form of expression. CMTM7 is widely expressed in human normal tissues, especially in immune cells while its protein is mainly located in cytoplasm and cell membrane. Previous studies have found that CMTM7 is frequently downregulated or absent in some cancers, partly because of the aberrant promoter CpG methylation and loss of heterozygosity. Overexpression of CMTM7 could inhibit cancer cells (KYSE410 and KYSE180) growth by inducing G1/S phase arrest and repressing EGFR-PI3K/AKT signaling in KYSE180 cells (92). The expression of CMTM7 could be dynamically adjusted by transcription factor FLI1 and SOX10 during tumor pathogenesis (93, 94). Another study indicated that the silence of CMTM7 could decrease Rab5 activation, promoting tumor growth and migration in non-small cell lung cancer (95). Huang et al. investigated the expression, function, and mechanism of CMTM7 in HCC through IHC and *in vitro* cell experiments (39). They found CMTM7 was significantly downregulated in HCC tissues and cell lines (Hep3B, SK-HEP-1, Huh7, and HepG2), and exhibited tumor-suppressor activities. CMTM7 is negatively correlated with the TNM staging of HCC and tumor metastasis. They further revealed that overexpression of CMTM7 could induce cell cycle arrest in G0/G1 phase by downregulation of cyclin D1 and cyclin-dependent kinase 4/6 (CDK4/6) expressions and upregulation of p27 expression, then inhibit the HCC cells growth and migration. Therefore, CMTM7 may serve as a potential biomarker to predict the possible of HCC invasion and metastasis. But similar with CMTM4, the results of the Human Protein Atlas are contrary, which showed that high CMTM7 expression is associated with poor prognosis (**Figure 1**). The causes of this phenomenon have yet to be elucidated, and should be explored in the future studies.

### CMTM8

CMTM8 has high similarity to TM4SF in the CMTM family, with 39.3% homology (2). Bioinformatics analysis reveals that CMTM8 is conserved during evolution, the similarities between human and mouse CMTM8 is 95.4% (2). CMTM8, the full-length cDNA product, is the predominant isoform and expresses in many cell lines and normal tissues. Jin et al. revealed that CMTM8 inhibited tumor cells (HEK293, HeLa and PC3) growth by accelerating the internalization of EGFR to attenuate EGFR-mediated signaling pathway (96). Further study demonstrated that CMTM8 induced cell apoptosis through both caspase-dependent and -independent pathway (97). Li et al. reported CMTM8-v2 as an alternative spliced isoform of CMTM8 to maintain the ability to induce cell apoptosis (98). But it doesn’t affect EGFR internalization because of lacking second exon which codes MARVEL domain and cytosolic YXXø motif. Previous studies have demonstrated that CMTM8 was frequently downregulated or silenced in multiple solid tumors (liver, lung, colon, rectum, esophagus, stomach, bladder, bone) (99–101). Overexpression of CMTM8 in bladder cancer can inhibit cell growth, migration and invasion both *in vivo* and *in vitro* (100). Downregulation of CMTM8 induced EMT-like processes *via* HGF/c-MET/ERK signaling in HCC cells (HepG2) and other epithelial cells, indicating CMTM8 was a key in regulating cell motility and invasion (40). Fewer study on the relationship between CMTM8 and HCC patient survival have been reported to date, but the Human Protein Atlas showed that CMTM8 is not a prognostic index in HCC (**Figure 1**). Therefore, more studies are needed to examine the real clinical significance of CMTM8 in HCC.

## Conclusions and Prospects

**Figure 3:**
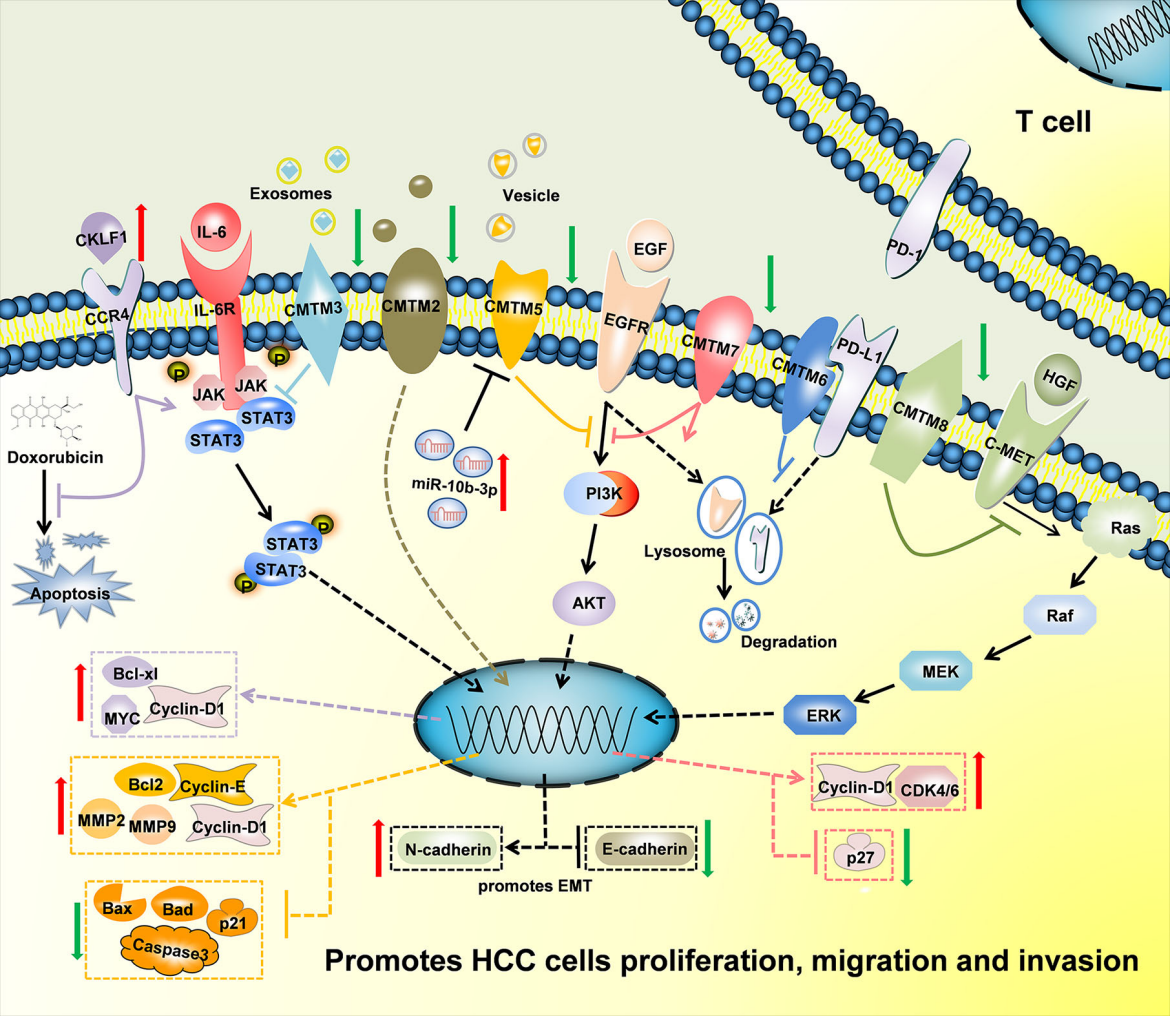
Schematic of the mechanisms regulated by CMTMs in Hepatocellular Carcinoma. CKLF1 enhanced hepatocarcinogenesis and prevented Doxorubicin-induced apoptosis *via* IL-6/STAT3 signaling pathway. Down-Regulated CMTM2, CMTM3, CMTM5, CMTM7 and CMTM8 promoted cells proliferation, migration, invasion, and induced epithelial-mesenchymal transition in Hepatocellular Carcinoma *via* JAK2/STAT3, PI3K/AKT, and c-MET/ERK signaling pathways. CMTM6 suppressed T-cell by stabilizing PD-L1 in the membrane. The mechanism of CMTM1 and CMTM4 is not clear yet.

In conclusion, CMTMs have different expression profiles in HCC and normal tissues (**Figure 2**). Individual members of the CMTM family play different roles in the development and progression of HCC, mainly involved in the cellular proliferation, apoptosis, metastasis and invasion (**Figure 3**). CKLFs promoted carcinogenesis and enhanced the ability to resist chemotherapy in HCC. Current studies showed that CMTM2-8 act as tumor suppressors in HCC and the negative expression are risk factors for poor prognosis of HCC. These findings truly exist, but the interesting distinctions found between these findings and the results of database make them become controversial. The anti-tumor effect mainly relate with the regulation of cell cycle, inhibition of the EGFR-induced cell growth and the EMT process. DNA methylation and/or microRNAs regulation of these CMTMs is the dominant mechanism of HCC to evade anticancer effect. Small molecular agonist/inhibitor of CMTMs might be a kind of treatment method in the future. Additionally, CMTM4 and CMTM6, as PD-L1 protein regulators, are expected to be potential immunotherapy targets of HCC. It’s still unknown whether CMTM1 displays positive or negative effect on HCC. Hence, further exploration of CMTMs expression, molecular mechanisms and related signaling pathways in HCC is necessary. The CMTM family could provide new ideas and targets for HCC diagnosis and treatment.

## Author Contributions

ML and FL collected related papers and drafted the manuscript. ML drafted the figures. XT and SY participated in the design of the review. LZ and SZ were responsible for the supervision of the work. All authors contributed to the article and approved the submitted version.

## Funding

Innovative Research Groups of National Natural Science Foundation of China (No. 81721091), National S&T Major Project (No. 2017ZX10203205), Zhejiang International Science and Technology Cooperation Project (No. 2016C04003), Research Unit Project of Chinese Academy of Medical Sciences (2019-I2M-5-030), and Grant from Health Commission of Zhejiang Province (JBZX-202004).

## Conflict of Interest

The authors declare that the research was conducted in the absence of any commercial or financial relationships that could be construed as a potential conflict of interest.
